# Metabolic Responses to Reductive Stress

**DOI:** 10.1089/ars.2019.7803

**Published:** 2020-05-14

**Authors:** Wusheng Xiao, Joseph Loscalzo

**Affiliations:** Division of Cardiovascular Medicine, Department of Medicine, Brigham and Women's Hospital, Harvard Medical School, Boston, Massachusetts, USA.

**Keywords:** NAD(H), NADP(H), GSH/GSSG, reductive stress, cellular metabolism, redox

## Abstract

***Significance:*** Reducing equivalents (NAD(P)H and glutathione [GSH]) are essential for maintaining cellular redox homeostasis and for modulating cellular metabolism. Reductive stress induced by excessive levels of reduced NAD^+^ (NADH), reduced NADP^+^ (NADPH), and GSH is as harmful as oxidative stress and is implicated in many pathological processes.

***Recent Advances:*** Reductive stress broadens our view of the importance of cellular redox homeostasis and the influences of an imbalanced redox niche on biological functions, including cell metabolism.

***Critical Issues:*** The distribution of cellular NAD(H), NADP(H), and GSH/GSH disulfide is highly compartmentalized. Understanding how cells coordinate different pools of redox couples under unstressed and stressed conditions is critical for a comprehensive view of redox homeostasis and stress. It is also critical to explore the underlying mechanisms of reductive stress and its biological consequences, including effects on energy metabolism.

***Future Directions:*** Future studies are needed to investigate how reductive stress affects cell metabolism and how cells adapt their metabolism to reductive stress. Whether or not NADH shuttles and mitochondrial nicotinamide nucleotide transhydrogenase enzyme can regulate hypoxia-induced reductive stress is also a worthy pursuit. Developing strategies (*e.g.*, antireductant approaches) to counteract reductive stress and its related adverse biological consequences also requires extensive future efforts.

## Introduction

Mammalian cells depend on a series of oxidation and reduction (redox) reactions to generate energy (*e.g.*, ATP) and to synthesize essential cellular components (*e.g.*, nucleic acids) from nutrients in support of their biological functions. In a redox reaction, electrons flow from reducing agents (reductants) to oxidizing agents (oxidants). Cellular redox couples, mainly nicotinamide adenine dinucleotide (NAD^+^)/reduced NAD^+^ (NADH), phosphorylated NAD^+^ (NADP^+^)/reduced NADP^+^ (NADPH), and reduced glutathione (GSH)/GSH disulfide (GSSG), are responsible for the bulk of cellular electron transfer (88, 106). These redox couples serve as cofactors or substrates for enzymatic or nonenzymatic neutralization of reactive oxygen species (ROS) to sustain a relatively reducing environment in cells.

Importantly, these redox couples also link the cellular redox environment with cellular energetics. For instance, NAD^+^ serves as an electron sink to support glycolysis; NADH provides electrons for mitochondrial oxidative phosphorylation (OXPHOS); and NADPH is a major electron source for reductive biosynthesis of fatty acids and nucleic acids (112, 115). Given that these reducing equivalents are indispensable for cellular redox homeostasis and energy metabolism, an imbalance of the redox state of these molecules is implicated in a variety of pathological conditions, such as cardiovascular diseases, neurodegenerative diseases, cancer, and aging (10, 115).

In this review, we first introduce the cellular redox network and redox stress, and then delineate the metabolic sources and cellular distribution of the NAD^+^/NADH, NADP^+^/NADPH, and GSH/GSSG redox couples. This information is followed by an explanation of how perturbations in these ratios lead to reductive stress. Finally, we discuss metabolic responses to reductive stress and their impact on cell function.

## Cellular Redox Network and Redox Stress

### Redox network in mammalian cells

**FIG. 1.**
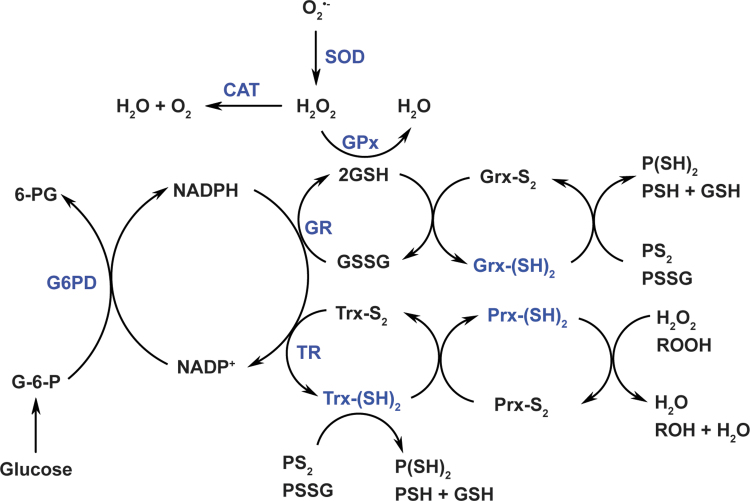
**Cellular redox network.** Cellular redox homeostasis is maintained by a delicate balance between pro-oxidant and antioxidant systems. Cellular pro-oxidants primarily include O_2_^•−^ and H_2_O_2_, which are generated by enzymes or/and mitochondrial respiration. SODs convert O_2_^•−^ to H_2_O_2_, which is further neutralized to water by catalase, GPxs, and Prxs. Catalase has very low affinity for H_2_O_2_ (but with a high turnover and high catalytic efficiency) and can reduce H_2_O_2_ to water when its levels reach the millimolar range. GPxs require two molecules of reduced GSH for H_2_O_2_ reduction and GSH is oxidized to GSSG in this reaction. GSH can also be used by glutaredoxins [Grx-(SH)_2_] to reduce protein intra/inter-disulfide (PS_2_ or PSSG) into reduced protein cysteinyl residues (P[SH]_2_ or PSH + GSH). The recycling of GSSG to GSH is catalyzed by GR, which utilizes NADPH as an electron donor. Prxs extract electrons from reduced thioredoxins [Trx-(SH)_2_] to reduce H_2_O_2_ or organic hydroperoxides (ROOH) to H_2_O or/and alcohols (ROH), respectively; and Trx-(SH)_2_ is simultaneously oxidized into Trx-S_2_. Like GSH, Trx-(SH)_2_ can also reduce protein disulfides into protein cysteinyls. The recycling of Trx-S_2_ is catalyzed by TRs using NADPH as an electron donor. NADPH is generated by G6PD in the PPP of glucose metabolism. Therefore, the cellular redox state and cell metabolism are closely linked through NADPH. Adapted from references (7, 8) with permission. 6-PG, 6-phosphogluconate; G-6-P, glucose-6-phosphate; G6PD, glucose-6-phosphate dehydrogenase; GPxs, glutathione peroxidases; GR, glutathione reductase; Grx, glutaredoxin; GSH, glutathione; GSSG, GSH disulfide; H_2_O_2_, hydrogen peroxide; NADPH, reduced NADP^+^; O_2_^•−^, superoxide anion; PPP, pentose phosphate pathway; Prxs, peroxiredoxins; PSSG, protein-glutathione disulfide; SODs, superoxide dismutases; TRs, thioredoxin reductases; Trx, thioredoxin.

The cellular redox niche is balanced by pro-oxidants and antioxidants. Cellular pro-oxidants include ROS (*e.g.*, superoxide anion [O_2_^•−^] and hydrogen peroxide [H_2_O_2_]), reactive nitrogen species (RNS; *e.g.*, nitric oxide [NO^•^]), and their derivatives (*e.g.*, peroxynitrite [ONOO^−^]), which are primarily produced by enzymes, such as NAD(P)H oxidases (NOXs) and uncoupled nitric oxide synthases, or by the mitochondrial electron transport chain as functional by-products (30, 87). To counteract these oxidants, cells have developed antioxidant systems, including antioxidant enzymes, for example, superoxide dismutases (SOD1–3), catalase, glutathione peroxidases (GPx1–8), thioredoxins (Trx1–2), and peroxiredoxins (Prx1–6); and nonenzymatic small molecules, for example, GSH, α-tocopherol, and ascorbate (7, 30, 87) (Fig. 1).

Of note, the production of cellular oxidants is compartment specific, but their targets can be local or/and remote from the source compartment. For example, mitochondria-generated H_2_O_2_ can function as a signaling molecule in mitochondria (local) or/and in cytosol (remote) (26). In the vasculature, endothelial cell-derived NO^•^ can diffuse from the endothelium to adjacent smooth muscle cells leading to vasodilation (22). Unlike oxidants, antioxidant enzymes exercise their functions depending on their specific subcellular localization. For example, manganese SOD (SOD2), a mitochondrial matrix-localized enzyme, can only convert O_2_^•−^ into H_2_O_2_ in mitochondria, where H_2_O_2_ is subsequently reduced to water by mitochondrial peroxidases (such as catalase, GPx1, GPx4, and Prx2).

The GSH/GSSG couple and the NADP^+^/NADPH couple play crucial roles in the two-electron reduction of peroxides. GSH is a cosubstrate of GPxs for H_2_O_2_ removal. In this context, two molecules of GSH donate two electrons to reduce H_2_O_2_ to water and themselves are oxidized to GSSG, which is then recycled back to GSH by glutathione reductase (GR) using NADPH as an electron donor (7, 8) (Fig. 1). It is interesting to note that glutaredoxins (Grx1–2) also utilize GSH to reduce protein intra/inter-disulfides (PS_2_ or protein-glutathione disulfide [PSSG]) into protein sulfhydryl groups (-SH) (7, 8) (Fig. 1). In addition to GR, NADPH is also an indispensable cofactor for thioredoxin reductases (TRs). Likewise, NADPH provides two electrons for TRs to reduce oxidized Trx (Trx-S_2_) to its active dithiol form Trx-(SH)_2_. The reduced form Trx-(SH)_2_ serves as an electron source for regeneration of Prxs, which reduce H_2_O_2_ and other organic hydroperoxides to water and alcohols, respectively (7, 8) (Fig. 1). Notably, intracellular total Trx content was estimated to be 1–10 μ*M* in bovine tissues (34), which is 2–3 orders of magnitude lower than total GSH levels (m*M* range).

### Redox stress

**FIG. 2.**
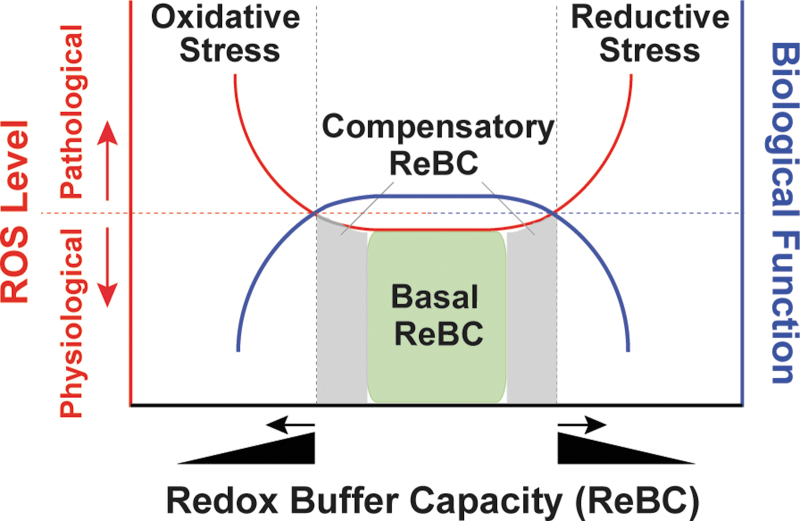
**Redox stress.** The NADH/NAD^+^, NADPH/NADP^+^, and GSH/GSSG redox couples are the major cellular redox buffers. Under unstressed conditions, these three redox couples have adequate capacity to maintain redox homeostasis, termed basal ReBC (X-axis; *green box under red line*). When cellular ROS levels increase (*Left* Y-axis; *red line*), these redox buffers are also able to respond by elevating basal ReBC to a certain level, termed the compensatory ReBC (X-axis; *gray area under red line*). Under both circumstances, cellular ROS levels are maintained at physiological levels to ensure normal biological function, such as signaling molecule adequacy (*Right* Y-axis; *blue line*). However, when this compensatory response continues beyond a certain threshold at which the maximal ReBC is exceeded by cellular reducing of ROS levels (X-axis, *black triangle*), reductive stress can occur (*red line*). By contrast, oxidative stress occurs when cellular ReBC decreases (X-axis, *black triangle*), or/and cellular oxidation of ROS production is overwhelming. Both reductive stress and oxidative stress (collectively named as redox stress) can promote ROS production leading to oxidative damages to macromolecules and perturbations of cellular functions. NAD^+^, nicotinamide adenine dinucleotide; NADH, reduced NAD^+^; NADP^+^, phosphorylated NAD^+^; ReBC, redox buffer capacity; ROS, reactive oxygen species.

Under physiological conditions, cellular pro-oxidants are maintained at steady-state levels required for many biological processes, such as cell signaling, proliferation, and differentiation (30, 87). However, when the balance between pro-oxidants and antioxidants is adversely perturbed, redox stress, including oxidative stress and reductive stress, occurs (Fig. 2).

The concept of oxidative stress was first introduced by Paniker *et al.* in 1970 in studies of the GSH/GSSG couple in H_2_O_2_-stimulated normal and GR-deficient human erythrocytes (74). This term is now defined as the imbalance between cellular pro-oxidant levels and antioxidant capacity in favor of the former (30, 52, 87), implying that an excess in oxidant levels leads to oxidative stress (Fig. 2). It is commonly accepted that oxidative stress induced by excess ROS/RNS levels is detrimental to biological functions and is involved in the development of many pathological conditions, such as cardiovascular diseases, neurodegenerative diseases, aging, and cancer (30, 90, 106); however, it is important to note that these oxidants (*e.g.*, O_2_^•−^ and H_2_O_2_) are capable of either oxidizing or reducing other molecules depending on the standard redox potential (E°′) of two reactants (Table 1) (89). For example, in the Fenton reaction, O_2_^•−^ is oxidized to O_2_ and H_2_O_2_ is reduced to hydroxyl radical (HO^•^), which is the most oxidizing species (E°′ = 2310 mV).

**Table 1.** Standard Reduction Potentials (E°′) of Representative Redox Couples

Redox couples	E°′/mV at 25°C	Reduction types	Oxidizing ability
NAD^+^, H^+^/NADH	−316	Two-electron reduction	Low High
NADP^+^, H^+^/NADPH	−315		
GSSG, 2H^+^/2GSH	−240		
Trx-S_2_, 2H^+^/Trx-(SH)_2_	−240		
Cys-S-S-Cys, 2H^+^/2Cys-SH	−230		
FMN, 2H^+^/FMNH_2_	−219		
FAD, 2H^+^/FADH_2_	−219		
Grx-S_2_, 2H^+^/Grx-(SH)_2_	−218		
Acetaldehyde, 2H^+^/alcohol	−197		
Pyruvate, 2H^+^/lactate	−183		
Oxaloacetate, 2H^+^/malate	−166		
2H_3_O^+^+2e^−^/H_2_(g), 2H_2_O	0		
Ubiquinone, 2H^+^/ubihydroquinone	+84		
Ascorbate^•−^, H^+^/ascorbate	+282	One-electron reduction	
O_2_, 2H^+^/H_2_O_2_	+300		
H_2_O_2_, 2H^+^/H_2_O, HO^•^	+320		
α-Tocopheroxyl^•^, H^+^/vitamin E	+500		
RS^•^/RS^−^ (thiolate)	+920		
O_2_^•−^, 2H^+^/H_2_O_2_	+940		
ROO^•^, H^+^/ROOH (alkylperoxyl radical)	+1000		
RO^•^, H^+^/ROH (aliphatic alkoxyl radical)	+1600		
HO^•^, H^+^/H_2_O	+2310		

Adapted from Schafer and Buettner (89) with permission.

GSH, glutathione; Grx, glutaredoxin; GSSG, GSH disulfide; H_2_O_2_, hydrogen peroxide; NAD^+^, nicotinamide adenine dinucleotide; NADH, reduced NAD^+^; NADP^+^, phosphorylated NAD^+^; NADPH, reduced NADP^+^; O_2_^•−^, superoxide anion; Trx, thioredoxin.

O_2_^•−^ + Fe^3+^ → O_2_ + Fe^2+^ (1)

H_2_O_2_ + Fe^2+^ → Fe^3+^ + HO^•^ + HO^−^ (2)

O_2_^•−^ + H_2_O_2_ → O_2_ + HO^•^ + HO^−^ (Net reaction)

Likewise, H_2_O_2_ can reduce ferryl hemoglobin and oxidize methionine; and itself is oxidized to O_2_^•−^ and reduced to water, respectively (52). Therefore, increases in O_2_^•−^ and H_2_O_2_ levels can be viewed as creating oxidative or reductive stress depending on the relative abundance of redox-coupled species.

Unlike oxidative stress, reductive stress is not as well-characterized a concept. This term was first described by Gores *et al.* in 1989, the experiments in which the authors mimicked anoxia by treating rat hepatocytes with chemicals to block mitochondrial respiration and ATP production (so-called chemical hypoxia) (27). The authors proposed that electron carriers became reduced due to limited oxygen availability (*e.g.*, hypoxia) and were reoxidized during reoxygenation (*e.g.*, reperfusion) leading to a burst of ROS generation, termed “reductive stress” (27). This term currently has a more generalized definition, that is, reductive stress is an imbalance between cellular pro-oxidant levels and reducing capacity in favor of the latter (30, 52, 87). In the context of this review, we define reductive stress as an excess accumulation of reducing equivalents (specifically NADH, NADPH, and GSH), exceeding the capacity of endogenous oxidoreductases.

Under physiological conditions, cellular redox buffers [GSH/GSSG and NAD(P)H/NAD(P)^+^] have sufficient capacity (termed basal redox buffer capacity [ReBC]) to maintain cellular oxidants and reductants at physiological levels (ROS as signaling molecules) (Fig. 2). When cells sustain oxidative or reductive insults, redox buffers increase to a certain level (termed compensatory ReBC) to counteract these redox stresses and restore redox homeostasis. Under these circumstances, cellular oxidants and reductants are still maintained within physiological ranges. However, when this compensatory response reaches a maximum, the ReBC is exceeded and oxidative or reductive stress occurs (Fig. 2). Importantly, reductive stress diminishes cellular ROS levels to below their physiological levels and thus perturbs their signaling functions. From a different viewpoint, reductive stress can also promote ROS production (*e.g.,* by partially reducing oxygen) and thus is proposed to promote “oxidative stress” in essence, depending on the redox couples in which these ROS are engaged (45, 92, 107).

Like oxidative stress, reductive stress also impairs cellular functions (31). For example, the endoplasmic reticulum (ER) has a relatively oxidizing environment, which is required for structural disulfide formation of membrane and secretory proteins. However, under reductive stress, protein disulfide bonds inappropriately form, resulting in activation of the unfolded protein response and ER stress (60). In line with this concept, we and others showed that reductive stress perturbs protein disulfide formation leading to ER stress in yeast and intracellular retention of membrane proteins in human cells (32, 60, 100, 113). Furthermore, mice with cardiac-specific overexpression of mutated human αB-crystallin (hR120GCryAB) displayed increases in GSH levels and in activities of GR, glucose-6-phosphate dehydrogenase (G6PD), catalase, and GPx (indicative of reductive stress), which led to the development of cardiac hypertrophy and heart failure in these mice (77, 78). Elevated cytosolic NADH/NAD^+^ and its resulting reductive stress were observed in early diabetic animals owing to increased oxidation of substrates (*e.g.*, sorbitol, nonesterified fatty acids, and glucuronic acid pathway metabolites), which was coupled with the reduction of NAD^+^ to NADH (38). Thus, reductive stress is deleterious to cells and has been linked to many pathological conditions, including cardiovascular diseases and diabetes mellitus (31, 51, 105).

## Metabolic Sources and Cellular Distribution of the NAD(H) and NADP(H) Couples

NAD^+^, a key cofactor for many enzymes including metabolic enzymes, is critical for various biological processes (106). Mammalian cells can synthesize NAD^+^ *de novo* from its precursors and recycle NAD^+^ from NADH. Depending on availability of its precursors, NAD^+^ can be synthesized *via* three pathways: the *de novo* pathway using tryptophan as a precursor, the *Preiss-Handler* pathway using nicotinic acid as a precursor, and the salvage pathway using nicotinamide (NAM) and nicotinamide riboside (NR) as precursors (106). NAD^+^ can be phosphorylated to NADP^+^ *via* NAD^+^ kinases (NADKs); the oxidized forms of NAD^+^ and NADP^+^ can be reduced to NADH and NADPH by their corresponding dehydrogenases (106). We and others have extensively reviewed the enzymatic steps and the regulatory factors of all three NAD^+^ biosynthetic pathways (106, 112, 114). In this review, we primarily focus on the interconversion of NAD(H) and NADP(H) *via* metabolic enzymes.

### Metabolic sources of the NAD(H) and NADP(H) couples

**FIG. 3.**
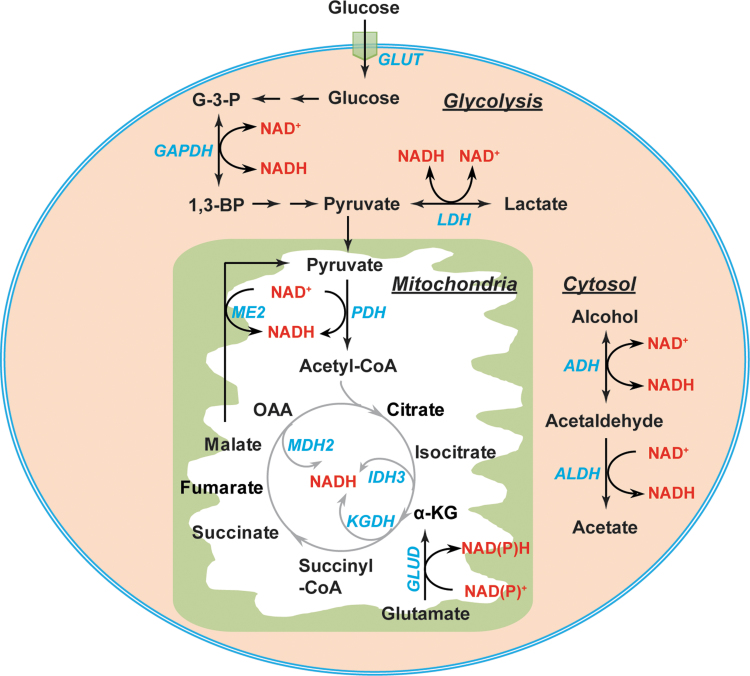
**Metabolic sources of NAD(H).** In the cytosol, the interconversion of NAD^+^ and NADH is mediated by two enzymes GAPDH and LDH and by the alcohol metabolism enzymes ADH and ALDH. In the mitochondrial matrix, PDH, ME2, GLUD, and TCA cycle enzymes (IDH3, KGDH, and MDH2) contribute to NAD(H) production. 1,3-BPG, 1,3-bisphosphoglycerate; α-KG, α-ketoglutarate; ADH, alcohol dehydrogenase; ALDH, aldehyde dehydrogenase; G-3-P, glyceraldehyde-3-phosphate; GAPDH, glyceraldehyde-3-phosphate dehydrogenase; GLUD, glutamate dehydrogenase; GLUT, glucose transporter; IDH, isocitrate dehydrogenase; KGDH, α-ketoglutarate dehydrogenase; LDH, lactate dehydrogenase; MDH2, malate dehydrogenase 2; ME, malic enzyme; OAA, oxaloacetate; PDH, pyruvate dehydrogenase; TCA, tricarboxylic acid.

**FIG. 4.**
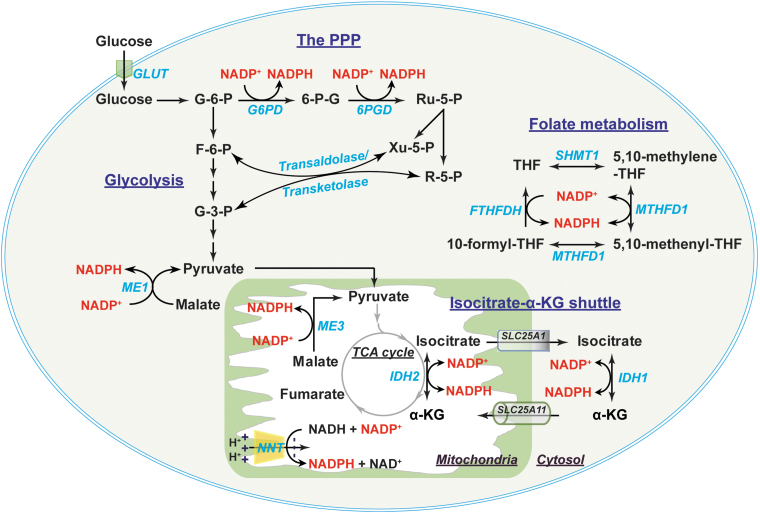
**Metabolic sources of NADP(H) and the NADPH shuttle.** In the cytosol, NADPH is primarily produced by G6PD and 6PGD in the PPP, where the products R-5-P and Xu-5-P can be diverted into glycolysis by transaldolase and transketolase. ME1 and IDH1 also contribute to cytosolic NADP(H) production. In addition, two enzymes in folate metabolism, MTHFD1 and FTHFDH, are also the sources of cytosolic NADP(H). Mitochondrial NADP(H) is generated by NADP^+^-dependent IDH2, NNT, and ME3. The cytosolic and mitochondrial NADP(H) pools are exchanged through the isocitrate-α-KG shuttle, where cytosolic IDH1 and mitochondrial IDH2 catalyze the interconversion of isocitrate and α-KG in conjunction with the interconversion of NADP^+^ and NADPH. The citrate carrier protein (encoded by *SLC25A1* gene) and the α-KG/malate antiporter (encoded by *SLC25A11* gene) mediate the transport of isocitrate and α-KG between the cytosol and mitochondria, respectively. 6PGD, 6-phosphogluconate dehydrogenase; FTHFDH, 10-formyl-tetrahydrofolate dehydrogenase; MTHFD, methylene-tetrahydrofolate dehydrogenase; NNT, nicotinamide nucleotide transhydrogenase; R-5-P, ribose-5-phosphate; Ru-5-P, ribulose-5-phosphate; SCL25A1, solute carrier family 25 member 1; SCL25A11, solute carrier family 25 member 11; SHMT, serine hydroxymethyltransferase; THF, tetrahydrofolate; Xu-5-P, xylulose-5-phosphate.

The interconversion of NAD(P)^+^ and NAD(P)H is mediated by metabolic enzymes involved in glycolysis, the pentose phosphate pathway (PPP), and the tricarboxylic acid (TCA) cycle (Figs. 3 and 4). Glycolysis and the TCA cycle are the two major sources of cytosolic and mitochondrial NAD(H), respectively (Fig. 3). In the cytosol, the interconversion of NAD^+^ and NADH is catalyzed by two enzymes, glyceraldehyde-3-phosphate dehydrogenase (GAPDH) and lactate dehydrogenase (LDH) (56, 106). GAPDH catalyzes the reversible conversion of glyceraldehyde-3-phosphate (G-3-P) to 1,3-bisphosphoglycerate in conjunction with the corresponding interconversion of the NAD^+^/NADH couple. LDH reduces pyruvate to lactate coupled with the oxidization of NADH to NAD^+^ in glycolysis. Of note, LDH can also catalyze the reverse reaction by reducing NAD^+^ to NADH and oxidizing lactate to pyruvate when lactate is utilized as a fuel source for the TCA cycle (37, 94). Additional sources of cytosolic NAD(H) include the alcohol dehydrogenases (ADHs) and aldehyde dehydrogenases (11).

In the mitochondria, eight molecules of NADH can be generated by per molecule of glucose through the TCA cycle under well-oxygenated conditions (112). The pyruvate dehydrogenase (PDH) complex reduces NAD^+^ to NADH in conjunction with decarboxylating pyruvate to acetyl-CoA. The latter enters the TCA cycle, where NADH is produced by isocitrate dehydrogenase 3 (IDH3), α-ketoglutarate dehydrogenase (KGDH), and malate dehydrogenase 2 (MDH2) (56, 106) (Fig. 3). Moreover, NAD^+^-linked malic enzyme (ME2) and glutamate dehydrogenases (GLUD1–2) also contribute to the mitochondrial NADH pool (81, 106) (Fig. 3).

Cytosolic NADPH is primarily generated from the PPP by G6PD and 6-phosphogluconate dehydrogenase (6PGD) (56, 106, 112). G6PD converts glucose-6-phosphate (G-6-P) to 6-phosphogluconate, which is further metabolized into ribulose-5-phosphate by 6PGD. Both reactions are coupled to the reduction of NADP^+^ to NADPH (Fig. 4). Of note, ribulose-5-phosphate and its derivative ribose-5-phosphate (R-5-P) can be converted into fructose-6-phosphate (F-6-P) and G-3-P by transaldolase/transketolase through a series of nonoxidative reactions and thereby be diverted into glycolysis (56).

In addition, other enzymes also contribute to the cytosolic NADPH pool, such as IDHs, MEs, methylene-tetrahydrofolate dehydrogenases (MTHFDs), and 10-formyl-tetrahydrofolate dehydrogenases (FTHFDHs) (56, 111), all of which have both cytosolic and mitochondrial isozymes. For example, cytosolic IDH (IDH1) and mitochondrial IDH2 catalyze the oxidation of isocitrate to α-ketoglutarate (α-KG) and the reduction of NADP^+^ to NADPH. Cytosolic malic enzyme (ME) 1 decarboxylates malate to pyruvate coupled with the reduction of NADP^+^ to NADPH. Moreover, two enzymes in folate metabolite also generate NADPH; cytosolic MTHFD (MTHFD1) catalyzes 5,10-methylene-tetrahydrofolate (5,10-methylene-THF) to 5,10-methenyl-THF; and cytosolic FTHFDH catalyzes the recycling of 10-formyl-THF to THF (111).

Mitochondrial NADPH can be produced by the mitochondrial enzymes IDH2, ME3, MTHFD2, and FTHFDH using the same reaction as their corresponding cytosolic isozymes (56, 111) (Fig. 4). NADP^+^-dependent GLUDs can also generate NADPH through the conversion of glutamate to α-KG (81). Of note, another significant source of mitochondrial NADPH is nicotinamide nucleotide transhydrogenase (NNT). NNT resides in the mitochondrial inner membrane and obtains electrons from NADH to reduce NADP^+^ into NADPH utilizing the proton gradient across mitochondrial inner membrane as a driving force: NADH + NADP^+^ ← → NAD^+^ + NADPH (35, 84). It has been estimated that IDH2, NNT, and ME3 contributed 70%, 22%, and 8%, respectively, of mitochondrial NADPH production in cardiac tissue of C57BL/6N mice (69).

### Compartmental distribution of cellular NAD(H) and NADP(H)

The intracellular distribution of NAD(H) and NADP(H) is highly compartmentalized owing to specific localization of their biosynthetic enzymes and membrane permeability to these dinucleotides (106). For example, mitochondrial nicotinamide mononucleotide adenylyltransferase and mitochondrial NADK support NAD^+^ and NADP^+^ synthesis, respectively, in this organelle (10, 71, 118). While NAD(P)^+^ can freely pass through the nuclear membrane, none of these dinucleotides can diffuse across the mitochondrial inner membrane (35, 114). Of note, different compartmental NAD(H) and NADP(H) pools differentially respond to exogenous stimuli. Typically, cytoplasmic and nuclear NAD(H) pools are more sensitive to changes in redox stress than the mitochondrial NAD(H) pool, which is stably maintained and required for cell survival under redox insults (108, 120).

Although cytosolic, nuclear, and mitochondrial NAD(H) and NAD(P)H pools often function in their respective compartments, these compartmental dinucleotide pools also exchange *via* shuttle mechanisms to maintain the overall cellular redox environment and redox-dependent functions. Cytosolic NADH can freely diffuse through the porous mitochondrial outer membrane; however, it is unable to pass through the mitochondrial inner membrane (10, 76, 112). To circumvent this limitation, cells developed two NADH shuttles: the malate/aspartate shuttle and the glycerol-3-phosphate shuttle (35, 65, 106).

In the first shuttle, cytosolic NADH is oxidized to NAD^+^ by MDH1 in conjunction with the reduction of oxaloacetate (OAA) to malate; whereas in the mitochondria, NAD^+^ is reduced to NADH by MDH2 coupled with the oxidation of malate back to OAA in the TCA cycle (106). This shuttle also requires two transporters, the α-KG/malate antiporter (encoded by *SLC25A11* gene), which transfers cytosolic malate into the mitochondrial matrix; and the aspartate/glutamate antiporter (encoded by *SLC25A13* gene), which transfers mitochondrial aspartate into the cytosol (106). Unlike the malate/aspartate shuttle, the glycerol-3-phosphate shuttle is irreversible and needs only one enzyme, glycerol-3-phosphate dehydrogenase (GPDH) (65). Cytosolic GPDH oxidizes NADH to NAD^+^ in conjunction with the reduction of dihydroxyacetone phosphate (DHAP) to glycerol-3-phosphate, whereas mitochondrial GPDH catalyzes the reverse reaction that oxidizes glycerol-3-phosphate into DHAP and reduces FAD into FADH_2_. Therefore, such shuttle mechanisms enable cells to maintain adequate NAD^+^ in the cytosol in support of glycolysis and sufficient NADH in the mitochondria in support of OXPHOS.

Like NADH, NADP(H) cannot diffuse across the mitochondrial inner membrane (10, 76, 112). The exchange of cytosolic and mitochondrial NADP(H) pools is, therefore, carried out by the isocitrate-α-KG shuttle through IDH1 and IDH2 isozymes (35, 73, 106) (Fig. 4). Cytosolic IDH1 and mitochondrial IDH2 catalyze the reversible interconversion between isocitrate and α-KG in conjunction with the interconversion of NADP^+^ and NADPH (106). This shuttle also requires two carrier proteins, the citrate carrier protein (encoded by *SLC25A1* gene), which exports mitochondrial isocitrate into the cytosol in exchange for malate; and the α-KG/malate antiporter, which is the same carrier as in the malate/aspartate shuttle (106). Thus, the isocitrate-α-KG shuttle plays a pivotal role in maintaining cellular NADPH levels. Taken together, these shuttle mechanisms enable cells to maintain redox and energy homeostasis under a wide range of physiopathological conditions.

## Biosynthesis and Cellular Distribution of GSH

GSH is the most abundant thiol antioxidant in mammalian cells. It serves as a substrate for H_2_O_2_ removal by GPxs and itself is oxidized to GSSG (55, 88). The recycling of GSSG to GSH is catalyzed by GR using NADPH as an electron donor. In addition to its antioxidant function, GSH is also involved in modulation of cellular redox signaling (*e.g.*, protein S-glutathionylation), regulation of cell proliferation and cell death, and detoxification of xenobiotics and their metabolites (2, 19, 55).

### GSH biosynthesis

**FIG. 5.**
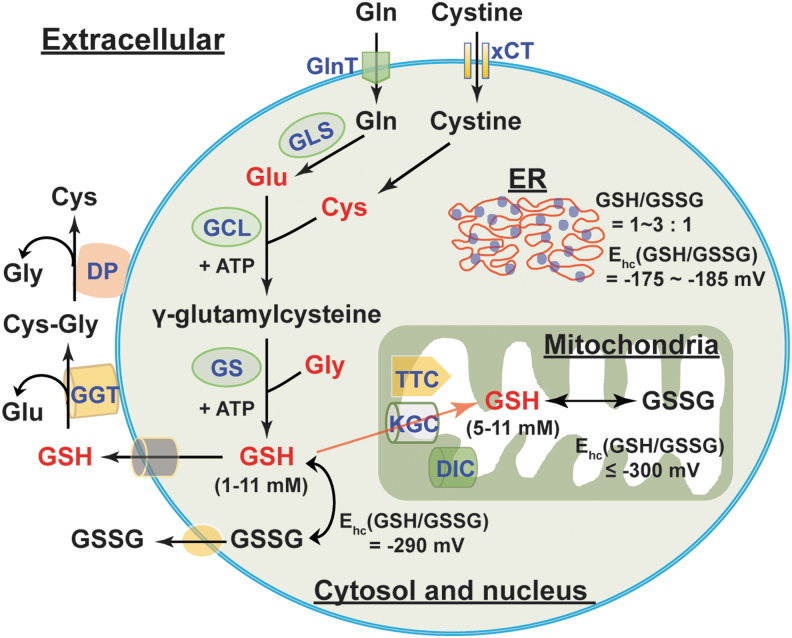
**The biosynthesis and cellular distribution of GSH.** Glu, Cys, and Gly are three precursor amino acids of GSH. GSH synthesis requires two successive steps catalyzed by two ATP-dependent enzymes: GCL (the rate-limiting enzyme) and GS. Glu can be replenished by endogenous and exogenous Gln *via* GLS-mediated glutaminolysis. Cys can be generated by reduction of endogenous and exogenous cystine. GSH levels in the cytosol and nucleus are within the 1–11 m*M* range with a half-cell reduction potential (E_hc_) of −290 mV. By contrast, the ER has a more oxidizing environment with GSH/GSSG of 1–3:1 and an E_hc_ of −175 to −185 mV. Cytosolic GSH can be transported into mitochondria through three potential transporters, KGC, DIC, and TTC. Mitochondrial GSH levels and the E_hc_ are comparable with that of cytosolic and nuclear pools. In addition, GSH and GSSG can also be secreted into the extracellular compartment; however, their levels are very low comparatively (within micromolar range). Extracellular GSH can be degraded by the cell surface enzyme GGT to form Glu and Cys-Gly, further metabolized into free Cys and Gly by DP. The resulting amino acids can be reused by cells for intracellular GSH synthesis. Cys, cysteine; Cys-Gly, cysteinyl-glycine; DIC, dicarboxylate carrier; DP, dipeptidases; ER, endoplasmic reticulum; GCL, γ-glutamylcysteine ligase; GGT, γ-glutamyl transpeptidase; GLS, glutaminase; Glu, glutamate; Gly, glycine; GS, GSH synthetase; KGC, α-KG carrier; TTC, tricarboxylate carrier; xCT, cystine transporter.

GSH is a tripeptide and is synthesized from precursor amino acids, l-glutamate, l-cysteine, and glycine, in the cytosol, through two ATP-dependent steps (Fig. 5). In the first step, glutamate reacts with cysteine to form γ-glutamylcysteine under the catalysis of the rate-limiting enzyme, γ-glutamylcysteine ligase (GCL). The GCL enzyme is composed of two functional subunits: catalytic (GCLC) and modifier (GCLM). In the following step, GSH synthetase (GS) catalyzes the formation of GSH by adding glycine to γ-glutamylcysteine (55, 88). Notably, glutamate and cysteine are linked by an unconventional γ-linkage peptide bond, which can be hydrolyzed only by γ-glutamyl transpeptidase (GGT) (2, 55). Since GGT is expressed on the exofacial surface of specific cells (*e.g.*, the liver, lung, vascular endothelium, kidney tubular cells, and biliary epithelial cells) (63), degradation of GSH takes place only in the extracellular space; intracellular GSH is resistant to degradation (2, 55).

The regulation of GSH synthesis is determined by the availability of its constituent amino acids, especially cysteine and glutamate, and the enzymatic activity of GCL. An excellent review by Lu in 2013 systematically summarized the mechanisms involved in modulations of cysteine availability and GCL activity (55). In the context of this review, we discuss recently identified mechanisms regulating cellular GSH levels.

Hypoxia-inducible factor 1α (HIF-1α) is a central transcriptional regulator of cellular metabolism under hypoxic conditions. Ample evidence supports that (pseudo)hypoxia increases cellular GSH levels *via* both HIF-1α-dependent and HIF-1α-independent mechanisms. Lu *et al.* reported that the *GCLM* subunit and the cystine transporter (xCT, encoded by *SLC7A11* gene) are transcriptional targets of HIF-1α in triple negative breast cancer cells under hypoxia (53). Treating these cells with chemotherapeutic drugs stimulated the mRNA and protein expression of these two genes and increased intracellular GSH levels through an HIF-1α-dependent mechanism (53). Knockout of prolyl hydroxylase domain-containing protein 2 *(PHD2)* stabilized HIF-1α protein and activated HIF-1α signaling, which transcriptionally upregulated glutaminase 1 (*GLS1*) expression leading to a decrease in ROS production and increases in cellular GSH levels, GSH/GSSG, and cell survival in murine periosteal progenitor cells, suggesting that HIF-1α signaling promotes glutaminolysis to provide glutamate for GSH synthesis (96).

Interestingly, HIF-1α-independent regulation of GSH synthesis has also been reported under hypoxia. Recent work showed that hypoxia upregulated xCT *SLC7A11* expression and increased intracellular cystine levels and GSH synthesis in multiple types of cancer cells (62, 82). Mechanistically, *SLC7A11* upregulation was mediated by activation of the protein kinase RNA-like ER kinase/eukaryotic initiation factor 2α (eIF2α) branch of the ER stress response but not HIF-1α signaling. Activation of transcription factor *eIF2α* could transcriptionally upregulate *SLC7A11* expression or posttranscriptionally stabilize *SLC7A11* mRNA in a cell type-specific manner (62, 82). In addition, p53 can transcriptionally upregulate mitochondrial glutaminase (*GLS2*) expression in several cancer cells under unstressed and stressed conditions (*e.g.*, ionizing radiation and H_2_O_2_) (36). Enhanced expression of *GLS2* by p53 activation or by ectopic overexpression promoted glutaminolysis leading to increases in cellular glutamate and GSH levels (36).

The metabolites lactate and fumarate are also regulators of GSH synthesis. For example, blockade of lactate export by inhibition of monocarboxylate transporter 1 increased intracellular lactate levels and decreased cellular γ-glutamylcysteine and GSH levels in human Raji Burkitt lymphoma cells (16). Mechanistically, lactate accumulation inhibited glucose uptake and glycolysis leading to depletion of cellular ATP levels. Since both GSH synthesis enzymes GCL and GS require ATP for activity, depletion of ATP by lactate suppressed activity and consequently GSH synthesis (16).

In addition, chronic accumulation of fumarate by deletion of fumarate hydratase (FH) led to the formation of succinic GSH, a covalent adduct of fumarate and GSH, which correlated with persistent oxidative stress and cellular senescence in renal cells (121). Interestingly, loss of FH also triggered a compensatory rise in GSH levels by upregulation of cystine transport *SLC7A11* expression and increases in cystine uptake and glutaminolysis-dependent GSH biosynthesis (121). Furthermore, evidence from diabetic rats and cultured mature adipocytes demonstrated that fumarate as an electrophile can directly react with GSH and cysteine residues in proteins *via* a Michael addition reaction forming S-(2-succinyl)-cysteine (succination), supporting the view that fumarate can irreversibly modify GSH (1, 67). Taken together, these lines of evidence highlight that GSH biosynthesis is influenced by several interrelated mechanisms, including transcriptional, posttranscriptional, and metabolic regulation.

### Cellular distribution of the GSH/GSSG couple

The GSH/GSSG couple is the principal redox buffer of a cell owing to its high concentration compared with other redox couples (88). Intracellular GSH distributes in different compartments (Fig. 5). The majority of intracellular GSH (>80%) is in the cytosol with an estimated concentration of 1–11 m*M*; mitochondrial GSH accounts for 10%–15% of the GSH, with its concentration estimated to be 5–11 m*M*. Five to 10% of total intracellular GSH is present in the nucleus with a concentration of the same or greater than cytosolic GSH; only a small percentage of GSH is in the ER (55, 88). Intracellular GSH is predominantly in its reduced form (>98%). The GSH/GSSG ratio in the cytosol is typically ≥30:1–100:1 with a redox potential of −290 mV; this ratio in the mitochondria is estimated to be >100:1 with a redox potential of ≤ −300 mV. By contrast, this ratio appears to be 1:1 to 3:1 in the ER with a redox potential of −175 to −185 mV, which accounts for the relatively oxidizing environment of the ER (9, 25, 75, 88).

It is notable that cytosolic GSH can exchange with other intracellular compartments. This compartmental exchange mechanism is essential for oxidative defense in the mitochondrion since it cannot synthesize GSH (54). GSH is a negatively charged molecule and cannot freely traverse mitochondrial membranes. Thus, the relocalization of cytosolic GSH to mitochondria requires specific carriers (Fig. 5). The α-KG carrier (encoded by *SLC25A11*) and the dicarboxylate transport mechanisms (dicarboxylate carrier, encoded by *SLC25A10*) are two potential candidates for importing cytosolic GSH to the mitochondria, mainly in the kidney and liver; the tricarboxylate carrier (encoded by *SLC25A1*) is a potential GSH transporter in the brain and astrocytes (9, 79). By contrast, some organelles, such as the nucleus, have their own GSH pools, which are independent of cytosolic GSH (88). This interesting phenomenon implies that cells likely preserve nuclear GSH to maintain a reducing environment and to prevent potential oxidative DNA damage. Thus, the distribution of intracellular GSH is organelle specific and exchange among different (but not all) pools is essential for maintenance of GSH homeostasis.

Mammalian cells can continuously secret GSH and GSSG into the extracellular compartment. *In vivo*, the liver is the major source of GSH in the circulation (55, 88). GSH levels in human plasma of healthy adults were estimated to be 2–5 μ*M* and the GSSG concentration was found to be much lower (0.14 μ*M*) (40, 66, 101). Intriguingly, plasma GSH levels declined in the elderly and in diabetic patients with a corresponding rise in GSSG levels (85), indicating a shift to a more oxidizing state in aging and diabetes. As mentioned above, extracellular GSH can be catabolized only in the extracellular space of certain cell types that express the GGT enzyme (2). GGT hydrolyzes GSH into glutamate and cysteinyl-glycine, which is further catabolized into cysteine and glycine by dipeptidases at the cell surface (2, 102) (Fig. 5). The resulting amino acids can be taken up by cells for intracellular GSH synthesis. Therefore, this extra/intercellular communication mechanism ensures cellular GSH levels are maintained optimally in the steady state.

## Reductive Stress Induced by NAD(P)H and GSH

As discussed in the Cellular Redox Network and Redox Stress section, NAD(H) can modulate energy metabolism; and NAD(P)H and GSH are indispensable reducing equivalents in response to oxidative stress. Paradoxically, excessive accumulation of cellular NAD(P)H and/or GSH leads to reductive stress and cellular dysfunction.

### NADH and reductive stress

**FIG. 6.**
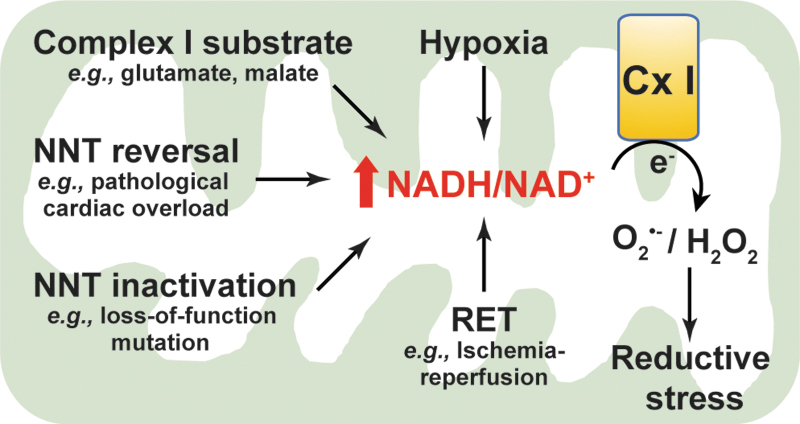
**Excess in NADH levels induces reductive stress.** Under stressed states, such as exogenous addition of Cx I substrates, hypoxia, NNT reversal, NNT inactivation, and RET, mitochondrial NADH/NAD^+^ increases leading to one-electron reduction of oxygen to O_2_^•−^ or/and two-electron reduction of oxygen into H_2_O_2_ at respiratory Cx I. Extensive reductive ROS levels result in reductive stress, which is detrimental to cellular function. Cx I, complex I; RET, reverse electron transfer.

An excess of NADH levels leads to reductive stress and ultimately ROS production (Fig. 6). Mitochondrial NADH is oxidized to NAD^+^ at mitochondrial respiratory complex I (NADH dehydrogenase). Emerging evidence supports that NADH oxidation at complex I is inhibited under hypoxia, which leads to a buildup of NADH and consequent reductive stress. Berney *et al.* reported that deletion of two [NiFe]-hydrogenases, *Hyd2* and *Hyd3*, increased the NADH/NAD^+^ ratio twofold under hypoxic conditions, resulting in reductive stress and cell death in oxygen-obligate mycobacteria (6). Likewise, hypoxia increased cytosolic NADH/NAD^+^ (indicated by an increase in lactate/pyruvate) and induced reductive stress, which correlated with ATP depletion and cytotoxicity in primary rat hepatocytes (43). Under hypoxia, accumulation of NADH is believed to provide more electrons for one-electron reduction of oxygen to O_2_^•−^ (14). Indeed, increases in cytosolic NADH/NAD^+^ and mitochondrial NAD(P)H levels were coupled with elevated mitochondrial ROS production in hypoxia-challenged bovine coronary artery smooth muscle cells (24). Recently, we reported that hypoxia triggered reductive stress and enhanced mitochondrial ROS generation in primary human lung fibroblasts (72).

In agreement with these *in vitro* studies, NADH-induced reductive stress and elevated mitochondrial ROS production were also observed in a cardiac ischemia/reperfusion (IR) injury mouse model. During ischemia, high NADH levels triggered a reversal of succinate dehydrogenase (SDH; complex II) activity leading to accumulation of succinate in various tissues, including the heart. After reperfusion, the accumulated succinate was rapidly reoxidized by SDH leading to ROS generation at complex I through a reverse electron transport mode and thereby cardiac IR injury (13). Blockade of ischemic succinate accumulation by inhibition of SDH attenuated mitochondrial ROS production and cardiac IR injury (13). Therefore, this evidence from various sources indicates that hypoxia increases the NADH pool leading to ROS generation and reductive stress.

In addition, reductive stress resulting from high NADH is also associated with elevated ROS production under ambient oxygen tensions. For example, addition of exogenous complex I substrates, such as glutamate plus malate, or α-KG, significantly augmented NADH levels and mitochondrial membrane potential, which stimulated H_2_O_2_ production by ∼10-fold in isolated rat brain mitochondria (95). Interestingly, this increase in H_2_O_2_ production was diminished by over 50% when NADH oxidation and mitochondrial respiration were enhanced by subsequent addition of ADP or the respiratory uncoupler carbonyl cyanide-p-trifluoromethoxy-phenylhydrazone (FCCP). Furthermore, subsequent addition of the complex I inhibitor rotenone entirely blocked NADH oxidation and caused a potentiation in H_2_O_2_ production after mitochondrial membrane potential was collapsed by addition of FCCP (95), indicating that a high NADH level is crucial for ROS production at complex I.

Likewise, in isolated bovine heart mitochondria, addition of NADH (1–3 μ*M*) promoted electron transfer from reduced flavin to O_2_ forming O_2_^•−^ at complex I within seconds (100–200 sec) (46). Treating rat L6 myoblasts with the antioxidant *N*-acetyl-l-cysteine (NAC; 1 m*M* for 1 h) induced reductive stress by increasing the NADH/NAD^+^ ratio, mitochondrial H_2_O_2_ levels, and free radical leak (93). In addition, insulin stimulation increased cellular lactate production and lactate/pyruvate (proportional to NADH/NAD^+^), which promoted cellular O_2_^•−^ production by NADH-dependent NOX enzymes in cultured SD rat thoracic aortic smooth muscle cells (110). Pretreatment with pyruvate or OAA significantly mitigated insulin-induced reductive stress in these cells. These cell-free and cell culture studies suggest that a high NADH/NAD^+^ promotes ROS production and induces reductive stress.

The mitochondrial NNT enzyme catalyzes reversible interconversion of the NADH/NAD^+^ and NADPH/NADP^+^ redox couples. Mounting evidence demonstrates that dysfunction of NNT leads to reductive stress. The NNT enzyme has a loss-of-function variant in C57BL/6J mice owing to a spontaneous mutation at the first exon and a multiple exon deletion (exon 7–11) in the *NNT* gene (99). Compared with NNT wild-type mice (C57BL/6N mice), isolated liver mitochondria from C57BL/6J mice had higher NADH/total NAD(H) and lower NADPH/total NADP(H) on ADP stimulation, which correlated with a reduced rate of removal of exogenous organic peroxides (33, 81).

Similarly, in isolated gastrocnemius muscle fibers of C57BL/6J mice, addition of pyruvate increased NADH production by the PDH complex, which correlated with a twofold elevation in H_2_O_2_ levels (17). The production of H_2_O_2_ was further augmented by fivefold when PDH complex flux was enhanced by the combination of pyruvate and carnitine (17). By contrast, H_2_O_2_ production was not affected in isolated muscle fibers from C57BL/6N mice (functional NNT) when flux from the PDH complex was stimulated by the same substrates (17). These results suggest that NNT loss-of-function leads to reductive stress through increasing NADH accumulation and decreasing NADPH production, and that NNT can integrate with the PDH complex to form a redox circuit regulating H_2_O_2_ production. In addition, knockdown of NNT increased cellular and mitochondrial NADH/total NAD(H) in human SK-Hep1 cells under basal and ADP-stimulated conditions compared with control shRNA-transfected cells, whereas cellular and mitochondrial NADPH/total NADP(H) was much lower in NNT-silenced cells, which correlated with an increase in mitochondrial ROS generation (33). These findings suggest that NNT loss-of-function blocks the interconversion of NADH to NADPH leading to NADH accumulation and reductive stress.

These observations were further supported by an *in vivo* study (69). Nickel *et al.* showed that under pathological cardiac pressure overload induced by transverse aortic constriction, NNT wild-type C57BL/6N mice displayed more severe heart failure and higher mortality compared with C57BL/6J mice lacking functional NNT activity (69). Enhanced failure in the myocardium of wild-type mice coincided with a decline in NADPH levels and an increase in NADH, H_2_O_2_ production, and oxidative damage (69). These findings suggest that pathological cardiac pressure overload induces NNT reverse functional mode to generate NADH at the expense of NADPH resulting in a high NADH/NAD^+^ and reductive stress, further promoting ROS at complex I leading to oxidative injury and cell death (Fig. 6).

Of note, the NADH/NAD^+^ imbalance-induced reductive stres1s is a common phenomenon in diabetes and diabetic syndromes. The central hypothesis is that diabetic hyperglycemia causes a pseudohypoxic increase in the NADH/NAD^+^ ratio and reductive stress (57, 103). Specifically, during chronic hyperglycemia, NADH production is enhanced through glycolysis and the polyol pathways, whereas NAD^+^ levels are depleted owing to overactivation of NAD^+^-consuming enzymes (*e.g.*, poly ADP ribose polymerase [PARP]), together leading to an increase in the NADH/NAD^+^ ratio and promoting ROS production by mitochondrial respiratory complex I due to NADH overload, eventually resulting in reductive stress (38, 105, 107). For example, using a streptozotocin-induced diabetic rat model, Wu *et al.* found activation of the polyol pathway and an upregulation of PARP-1 in the lungs of diabetic rats compared with nondiabetic animals, which correlated with increases in the NADH/NAD^+^ ratio, the activities of mitochondrial respiratory complex I–IV, and cellular ROS production (104), supporting the view that reductive stress occurs in the development of diabetic complications. Taken together, these lines of *in vitro* and *in vivo* evidence highlight that excess NADH induces reductive stress and ultimately promotes ROS generation.

### NADPH/GSH and reductive stress

**FIG. 7.**
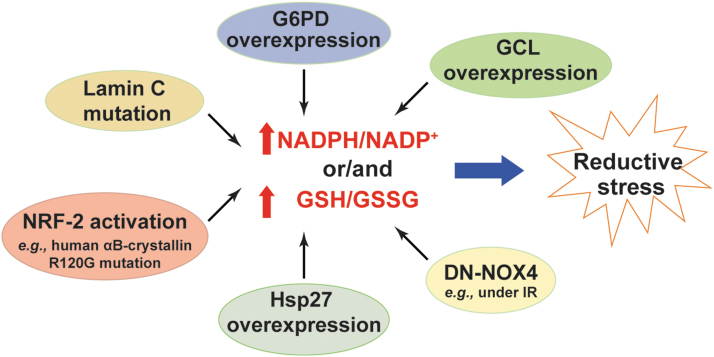
**Excess in NADPH or/and GSH levels triggers reductive stress.** Increases in NADPH/NADP^+^ or/and GSH/GSSG due to increases in their production (*e.g.*, G6PD overexpression, NRF-2 activation, Hsp27 overexpression, GCL overexpression, and lamin C mutation) or decreases in their consumption (*e.g.*, overexpression of DN-NOX4) lead to reductive stress. DN-NOX4, dominant negative NADPH oxidase 4; Hsp27, heat shock protein 27; IR, ischemia/reperfusion; NRF-2, nuclear factor (erythroid-derived 2)-like 2.

NADPH and GSH are essential for oxidative stress defense; and NADPH is indispensable for GSH recycling by GR (see the Cellular Redox Network and Redox Stress section). However, excessive levels of cellular GSH and/or NADPH also lead to reductive stress (Fig. 7).

NADPH is a substrate for NOX enzymes to produce H_2_O_2_ and O_2_^•−^ (5). As expected, cardiac-specific overexpression of wild-type *NOX4* (WT-NOX4) decreased NADPH/NADP^+^ and GSH/GSSG, which correlated with enhanced ROS production and cardiac dysfunction in mice under IR challenge (116). By contrast, overexpression of a dominant negative mutant of *NOX4* (DN-NOX4; loss of NOX4 activity) increased the ratios of these two redox couples in the myocardium. Paradoxically, elevated ROS levels and cardiac dysfunction were also observed in DN-NOX4 mice under IR challenge, indicating that high NADPH and GSH levels also enhance ROS generation and induce reductive stress by mechanisms yet to be determined (116).

G6PD is the major source of the cytosolic NADPH pool (see the Metabolic Sources and Cellular Distribution of the NAD(H) and NADP(H) Couples section). Overexpression of *G6PD* increased cellular NADPH levels and upregulated the expression of NOX cofactors, which potentiated ROS production and oxidative damage in mouse pancreatic β cells and thymic lymphoma cells (48, 97). By contrast, loss of G6PD activity was found to be beneficial in many disease models by limiting generation of high NADPH levels and reductive stress. For example, G6PD deficiency reduced NADPH levels and significantly inhibited angiotensin II (Ang II)-induced O_2_^•−^ production and thickening of the medial layer of the aorta (64). Similar effects were observed in pacing-induced heart failure where inhibition of G6PD activity abrogated elevations in NADPH levels and ROS production in failing hearts (28).

Furthermore, mice with cardiac-specific overexpression of a mutated human *αB-crystallin* (R120G mutant) developed protein aggregation cardiomyopathy and exhibited reductive stress in the heart as evidenced by increases in GSH levels, GSH/GSSG, and the activities of GR, G6PD, catalase, and GPx (77). Notably, these pathological alternations and reductive stress were significantly normalized by replacing the wild-type *G6PD* with a hypomorphic *G6PD* mutant in the R120G mice (77), suggesting that the G6PD-mediated reductive stress contributes to the development of this disease. Mechanistically, the reductive stress observed in R120G mutant mice was attributed to activation of the nuclear factor (erythroid-derived 2)-like 2 (NRF-2) signaling pathway, which transcriptionally upregulated the mRNA or/and protein expression of key antioxidant enzymes (*e.g.*, *GR*, *G6PD*, *GPx1*, and *catalase)* and GSH synthetic enzymes (*e.g.*, *GCLC* and *GCLM*), leading to increases in the GSH content and GSH/GSSG ratio, and to a decrease in ROS levels in heart tissue (77, 91). Importantly, knockout of *NRF2* significantly aborted the upregulated gene expression, normalized reductive stress, and reduced cardiac hypertrophy in R120G mutant mice (41, 78).

Likewise, a lamin C mutation in muscle tissues of *Drosophila* or human muscular dystrophy patients led to reductive stress demonstrated by increases in GSH and NADPH levels and activation of the NRF-2 pathway (15). Mechanistically, the increase in NADPH levels was contributed by elevated IDH activity but was independent of G6PD and 6PGD activity in this model (15). Unlike under oxidative stress where NRF-2 activation is mediated by oxidative inactivation of its sequester protein Keap-1, its activation under reductive stress was mediated by upregulation of an autophagy adaptor p62/SQSTM1, which also binds Keap-1 and thus enables nuclear translocation of NRF-2 and activation of its target genes (15).

In addition, transgenic mice with cardiac-specific heat shock protein 27 *(Hsp27)* overexpression developed cardiac hypertrophy and dysfunction, which correlated with increases in GSH levels, GSH/GSSG, and GPx1 activity and with a decrease in total ROS levels in the heart (119). Inhibition of GPx1 activity partially normalized the cardiomyopathy in Hsp27 transgenic mice, suggesting that reductive stress induced by excess GSH levels and GPx1 activity contributes to cardiac dysfunction in this model (119). Furthermore, increases in cellular GSH levels by either supplementing with the thiol-donor NAC or overexpressing GSH synthetic enzyme GCLC or/and GCLM subunit(s) diminished cellular ROS levels and reduced cellular 2GSH/GSSG redox potential by 7–12 mV (117). Paradoxically, this more reducing redox environment correlated with increases in mitochondrial oxidation and cell death, suggesting that elevated GSH levels result in reductive stress and cytotoxicity (117). Taken together, these lines of evidence clearly support that excess NADPH or/and GSH levels induce reductive stress.

## Metabolic Responses to Reductive Stress

The NAD(H), NADP(H), and GSH/GSSG redox couples are critical regulators of cellular metabolism. Specifically, glycolysis and the TCA cycle produce NADH, which provides electrons for mitochondrial OXPHOS and ATP production (56). NADP^+^ supports the PPP to generate NADPH that is indispensable for reductive biosynthesis of nucleotides, amino acids, and lipids (56). GSH links amino acid metabolism with cellular redox status. Thus, reductive stress induced by altered ratios of these three redox couples affects cellular metabolism and *vice versa*.

### Metabolic coordination under NAD(P)H-induced reductive stress

Metabolic adaption to hypoxia is a vital survival mechanism for metazoa. Under hypoxia, cell metabolism shifts away from mitochondrial OXPHOS to glycolysis for energy production, which is primarily mediated by activation of HIF-1α signaling. As mentioned in the Reductive Stress Induced by NAD(P)H and GSH section, hypoxia also induces reductive stress by NADH accumulation. Whether or not there is a link between reductive stress and metabolic reprogramming had not been well understood until recently.

We and others showed that l-2-hydroxyglutarate (L2HG), a reduced metabolite of α-KG by MDH1/2 or LDHA, selectively accumulated as a fundamental response to hypoxia in various primary and cancer cells (39, 72). The accumulation of L2HG levels correlated with increases in cellular NADH/NAD^+^ and mitochondrial ROS production indicative of reductive stress under hypoxia (72). Strikingly, increasing cellular L2HG levels by knockdown of L2HG dehydrogenase (the only known enzyme that oxidizes L2HG back to α-KG) decreased mitochondrial oxygen consumption and lactate production, which indicates that L2HG could inhibit both mitochondrial respiration and glycolysis to blunt their production of NADH and to mitigate attendant reductive stress (72).

The mitochondrial NNT enzyme is an important source of mitochondrial NADPH levels (see the Metabolic Sources and Cellular Distribution of the NAD(H) and NADP(H) Couples section). Overexpression of *NNT* increased NADPH/NADP^+^ resulting in reductive stress in melanoma cells (23). Intriguingly, NADPH-dependent reductive stress stimulated glutaminolysis to generate α-KG, which was then converted to succinyl-CoA by oxidative decarboxylation in the TCA cycle or to citrate *via* IDH2-mediated reductive carboxylation (23). By contrast, *NNT* silencing decreased NADH/NAD^+^ and NADPH/NADP^+^, which correlated with a decline in glutamine catabolism and a rise in the flux of glucose oxidation into the TCA cycle and energy production thereby (23). These results suggest that melanoma cells switch their energy source from glucose to glutamine under conditions of reductive stress.

A very recent study also reported reductive stress and metabolic reprogramming in human SK-Hep1 cells with *NNT* knockdown (33). Specifically, knockdown of *NNT* resulted in elevations in cellular and mitochondrial NADH/total NAD(H) and decreases in cellular and mitochondrial NADPH/total NADP(H) with a concomitant increase in mitochondrial O_2_^•−^ production, indicating that *NNT* silencing induces reductive stress in these cells (33). Intriguingly, *NNT* silencing also promoted mitochondrial membrane hyperpolarization, increased oxygen consumption and ATP production, and diminished lactate production, implying a metabolic shift from glycolysis to mitochondrial OXPHOS to correct the accumulation of NADH in these cells. This metabolic shift led these cells being more vulnerable to the cytotoxic effects of the mitochondrial OXPHOS inhibitor rotenone but more resistant to the glycolysis inhibitor 3-bromopyruvate (33). Moreover, enhanced glutaminolysis and reductive carboxylation were also observed in *NNT-*silenced cells, leading to accumulation of α-KG and its derivative metabolites (*e.g.*, citrate, fumarate, and malate). Mechanistically, this inhibition of glycolytic activity was attributable to destabilization of HIF-1α protein, possibly through α-KG-dependent PHD enzyme-mediated hydroxylation and enhanced degradation of HIF-1α protein (33).

In addition, primary mouse aortic endothelial cells isolated from C57BL/6J mice consumed less oxygen, had lower GPx activity, and produced more O_2_^•−^ when stimulated by Ang II compared with cells from C57BL/6N mice, suggesting that loss of NNT function impairs mitochondrial function (49). Furthermore, increasing evidence strongly supports that reductive stress observed in C57BL/6J mice lacking functional NNT correlated with systemic metabolic alterations demonstrated by glucose intolerance, reductions in glucose-induced insulin secretion and energy expenditure, and an increase in susceptibility to high-fat diet-induced obesity (17, 20, 21, 33, 68, 80).

Of note, NNT can also operate in a reverse mode to generate NADH from NADPH and NAD^+^, resulting in elevated NADH/NAD^+^, reductive stress, and metabolic adaption. For example, in C57BL/6N mice challenged with pressure overload using transverse aortic constriction, NNT operated through its reverse enzymatic mode, which promoted NADH production at the expense of NADPH and its antioxidant capacity (69). Increased NADH production accelerated mitochondrial respiration by fueling to mitochondrial OXPHOS, which combined with the compromised antioxidant capacity of NADPH resulted in increases in mitochondrial ROS production and oxidative damage, and ultimately cardiac dysfunction (69). These findings indicate that cardiomyocytes correct NADH-induced reductive stress by stimulating its oxidation through mitochondrial respiration.

In isolated pancreatic islets of C57BL/6N mice, glucose stimulation increased the NADPH/total NADP(H), which correlated with a reduction in mitochondrial GSH oxidation (86). Interestingly, the increased ratio was caused by a suppression of the reverse operation of NNT enzyme rather than a stimulation of its forward operation since NADH/total NAD(H) was also elevated by the identical challenge. The rise in NAD(P)H levels correlated with enhancement of glucose-stimulated insulin secretion, glycolysis (as demonstrated by elevated levels of G-6-P and F-6-P), and mitochondrial respiration (as demonstrated by increases in glucose oxidation, oxygen consumption, ATP production, and the TCA cycle intermediate metabolites citrate, malate, and fumarate) (86). Collectively, NNT is therefore a key enzyme bridging cell metabolism and reductive stress.

**FIG. 8.**
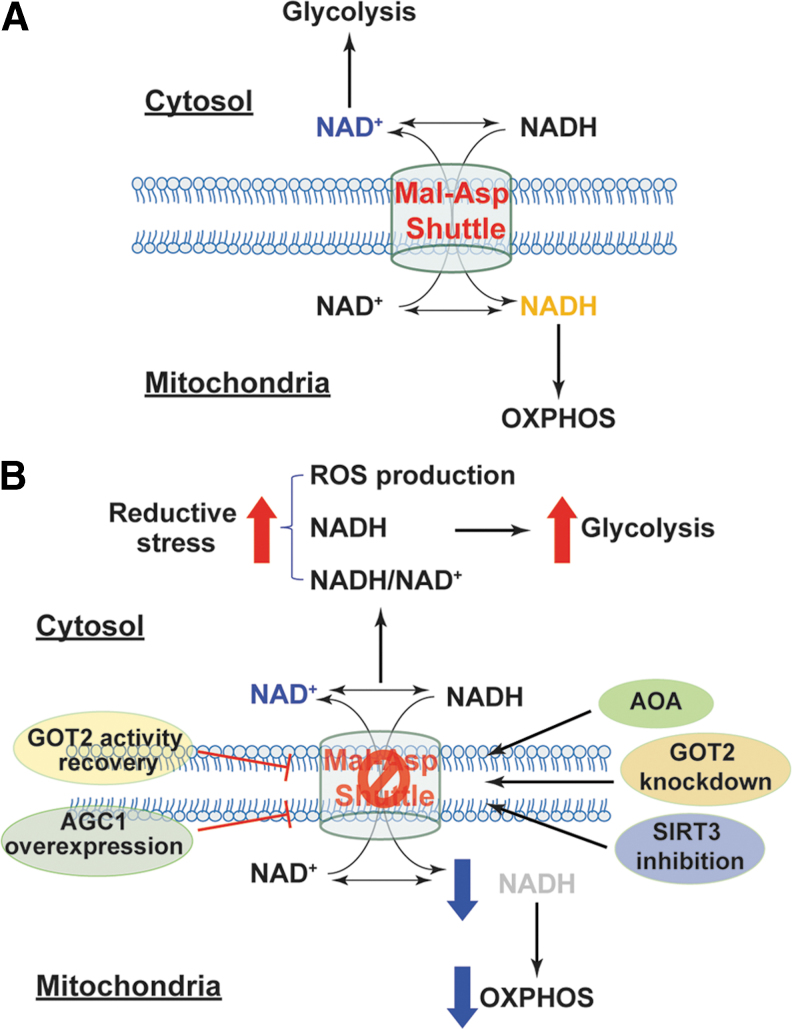
**Inhibition of the malate/aspartate NADH shuttle induces reductive stress and metabolic reprogramming. (A)** The malate/aspartate shuttle exchanges cytosolic NAD(H) with mitochondrial NAD(H) to maintain high cytosolic NAD^+^ levels, which are required for glycolysis, and high mitochondrial NADH levels, which provide electrons for mitochondrial OXPHOS. **(B)** Inhibition of the malate/aspartate shuttle by silencing *GOT2*, suppressing SIRT3 activity, or using the chemical inhibitor AOA leads to accumulation of cytosolic NADH and increases in cytosolic NADH/NAD^+^ and reductive ROS generation, indicative of reductive stress. NADH-induced reductive stress shifts cell metabolism from mitochondrial respiration to glycolysis. Enhancement of this shuttle activity by overexpression of *AGC1* or recovery of GOT2 activity significantly increases mitochondrial NADH levels and enhances mitochondrial respiration. AGC1, aspartate-glutamate carrier 1; AOA, aminooxyacetic acid; GOT2, glutamate-oxaloacetate transaminase; Mal-Asp shuttle, malate/aspartate shuttle; OXPHOS, oxidative phosphorylation; SIRT3, *sirtuin* deacetylase family member 3.

As discussed in the Metabolic Sources and Cellular Distribution of the NAD(H) and NADP(H) Couples section, the malate/aspartate shuttle is a pivotal mechanism for compartmental exchange of NADH and NAD^+^ to maintain high NAD^+^ levels in the cytosol and high NADH levels in mitochondria. Disruption of this exchange mechanism could perturb NADH/NAD^+^ in both compartments and thus could affect cellular redox homeostasis and energy metabolism (Fig. 8). For example, knockdown of mitochondrial glutamate-OAA transaminase (*GOT2*), a key enzyme in the malate/aspartate shuttle, increased cytosolic NADH levels and concurrently reduced mitochondrial NADH levels, which were concomitant with decreased NADPH/NADP^+^ and an increase in cellular ROS production in PANC-1 cells (109). *GOT2* silencing also significantly inhibited cellular ATP production and cell proliferation, indicating suppression of mitochondrial respiration (109). Interestingly, recovery of GOT2 activity by re-expressing an acetylation-mimetic *GOT2* in these cells inhibited cellular ROS production and restored mitochondrial NADH levels, cellular ATP production, and cell proliferation (109).

Moreover, inhibition of the malate/aspartate shuttle with aminooxyacetic acid (AOA, a potent inhibitor of aspartate aminotransferase) augmented cytosolic NADH/NAD^+^ reflected by elevated lactate/pyruvate and glycerol-3-phosphate/DHAP resulting in a more reductive environment in the cytoplasmic compartment of primary porcine aortic smooth muscle cells (3, 4). Notably, AOA treatment also attenuated glucose oxidation and oxygen consumption but increased glycolytic activity and lactate production, indicating a metabolic shift toward glycolysis (3, 4). These results suggest that perturbation of the malate/aspartate shuttle results in cytosolic retention of NADH and reductive stress, decreasing NADH availability in mitochondria and possibly increasing NAD^+^ levels in the cytosol, thereby leading to inhibition of NADH-dependent mitochondrial respiration and enhancement of NAD^+^-dependent glycolysis.

Such important roles of this shuttle have also been demonstrated *in vivo*. Cardiac-specific deletion of mitochondrial complex I subunit NDUFS4 protein resulted in accumulation of NADH in mitochondria and elevation in NADH/NAD^+^ in rodent heart, which correlated with the accelerated development of heart failure induced by pathological pressure overload or isoproterenol (42, 47). Intriguingly, isolated cardiomyocytes from these mice produced lower H_2_O_2_ and O_2_^•−^ than cells from wild-type mice (42, 47), indicating that complex I dysfunction leads to reductive stress in the failing heart. Since supraphysiological levels of NADH were reported to inhibit NAD^+^-dependent *sirtuin* deacetylase (SIRT1–7) (50), accumulated NADH in mitochondria repressed mitochondrial SIRT3 activity leading to hyperacetylation of mitochondrial proteins (42, 47) (Fig. 8). Specifically, the malate/aspartate shuttle proteins MDH2, α-KG/malate carrier, and GOT2 were found to be hyperacetylated leading to suppression of the transport and oxidation of cytosolic NADH in mitochondria, which correlated with decreases in oxygen consumption and cardiac energetics (manifested by a reduction in phosphocreatine/ATP) (42, 47). All of these pathological and biochemical changes were significantly abrogated by feeding the NAD^+^ precursor NAM mononucleotide to mice or overexpressing NAM phosphoribosyltransferase, a rate-limiting enzyme of NAD^+^ biosynthesis, in cardiac tissue (42, 47). Therefore, these results support that reductive stress induced by high NADH/NAD^+^ impairs mitochondrial function and energy metabolism in the heart.

By contrast, overexpression of human aspartate-glutamate carrier 1 (*AGC1*, encoded by *SLC25A12* gene) enhanced malate/aspartate shuttle activity on glucose stimulation in rat INS-1E β cells as demonstrated by increases in mitochondrial NAD(P)H levels and cellular glutamate levels (83) (Fig. 8). Moreover, *AGC1* overexpression also potentiated glucose-induced increases in glucose oxidation, mitochondrial membrane hyperpolarization, ATP production, and insulin secretion but inhibited lactate secretion compared with empty adenoviral vector-transfected cells (83), indicating that elevated NAD(P)H levels in mitochondria promote mitochondrial respiration and energy production. Collectively, these lines of evidence support the views that the malate/aspartate shuttle is essential for carrying electrons from cytosolic NADH to mitochondria for OXPHOS and that disruption of such a shuttle leads to reductive stress and metabolic dysfunction.

### Metabolic coordination under GSH-induced reductive stress

As discussed in the Biosynthesis and Cellular Distribution of GSH section, glutamate is a precursor amino acid of GSH. Glutamate is also a source of α-KG through glutaminolysis, where glutamine is hydrolyzed into glutamate by GLSs (cytosolic GLS1 and mitochondrial GLS2) followed by glutamate conversion into α-KG by GLUDs. Consequently, enhancing glutaminolysis could increase cellular GSH levels and alter cellular redox status and energy metabolism. For example, overexpression of *GLS2* markedly elevated cellular glutamate, GSH, and NADH levels as well as GSH/GSSG in three human cancer cell lines, which correlated with diminution in cellular basal ROS levels, implying reductive stress occurs in these cells (36). Interestingly, *GLS2-*overexpressing cells exhibited increases in cellular α-KG levels, mitochondrial oxygen consumption, and ATP production, suggesting that mitochondrial OXPHOS and the TCA cycle are enhanced under reductive stress. Importantly, reductive stress and metabolic stimulation were significantly normalized by silencing *GLS2* (36).

By contrast, GLS1 but not GLS2 was found to mediate HIF-1α-mediated enhancement of glutaminolysis in murine skeletal muscle cells. Activation of HIF-1α signaling by PHD2 deletion under normoxia enhanced glutamine uptake and upregulated *GLS1* expression leading to enhanced glutaminolysis and increases in cellular GSH levels and in GSH/GSSG, which correlated with upregulation of antioxidant genes (*SOD1*, *SOD2*, *catalase*, *GPx1*, and *GR*) and reduction in cellular and mitochondrial ROS production, indicatives of reductive stress (96). Interestingly, this reductive stress was accompanied by decreases in oxygen consumption and palmitate β-oxidation and increases in glycolytic activity and glycogen storage in these cells (96), indicating inhibition of mitochondrial function and stimulation of glycolysis and glycogenesis.

Furthermore, a recent study revealed that GSH is required for metabolic integration and reprogramming in murine T cells during inflammation (61). On activation, murine T cells expressed 6–10-fold higher *GCLC* mRNA than resting T cells, which was accompanied by elevated cellular GSH levels. Activated T cells reprogrammed their metabolism by enhancing fluxes of glutamine into the TCA cycle and of glucose into glycolysis in a c-MYC-dependent manner (61). These metabolic adaptive responses were effectively abolished in T cells with *GCLC* deletion and recapitulated by exogenous addition of GSH or its precursor NAC (61). Collectively, these findings underscore a close link between GSH-related reductive stress and energy metabolism.

## Concluding Remarks

The NAD(H), NADP(H), and GSH/GSSG redox couples are cofactors or/and substrates for many redox and metabolic enzymes. A delicate balance between the reduced and oxidized forms within each redox couple is a prerequisite for support of cellular redox homeostasis and energy metabolism. When that balance is lost, an excess in cellular NAD(P)H and GSH levels leads to reductive stress, metabolic stress, and cell dysfunction.

As an emerging concept, reductive stress complicates, but expands our understanding of the cellular redox environment. Traditionally, a reducing environment is thought to be beneficial for cell functions and biological processes since an oxidizing environment can lead to oxidative damage to proteins, lipids, and nucleic acids. Reductive stress highlights the facts that excess reducing equivalents (NAD(P)H and GSH) are also detrimental to cells *via* many mechanisms, including disruption of signaling functions of ROS, induction of ROS generation, perturbation of cell metabolism, and inhibition of protein disulfide formation. Thus, mammalian cells must maintain a balanced redox environment to avoid both oxidative and reductive damage.

Current knowledge about the underlying mechanisms of reductive stress and its biological consequences as well as how cells respond to reductive stress remains limited. For example, although it is clear that NADH accumulation under hypoxia induces reductive stress, it is uncertain as to (i) how NADH accumulates under hypoxia beside inhibition of its oxidation by mitochondrial respiration; (ii) whether or not NADH accumulation is compartment-specific; (iii) whether or not the two NADH shuttles are involved in redistribution; (iv) whether or not the NNT enzyme is involved in redistribution; (v) whether and how NADH accumulation affects the metabolic fates of glucose, lipids, and glutamine; and (vi) how cells respond to mitigate this insult. The answers to these questions remain largely open and require future experimental efforts.

In addition, the influences of GSH-induced reductive stress on cellular metabolism and *vice versa* are also open questions. This work summarizes limited information that is available in the literature on how modulation of GSH biosynthesis affects cellular metabolism. Notably, another important mechanism by which GSH could modulate cellular metabolism is through protein S-glutathionylation. Like phosphorylation, S-glutathionylation is an important posttranslational modification mechanism that regulates biological function, structural protein folding, and subcellular localization of a target protein. Proteins can be glutathionylated through both nonenzymatic and enzymatic reactions.

The former type of S-glutathionylation is primarily processed during oxidative stress and is often nonspecific and irreversible (58, 79). ROS oxidize GSH into GSSG, which reacts with cysteinyl residues (-SH) on proteins to form PSSG. Ample evidence demonstrates that metabolic enzymes are susceptible to glutathionylation under oxidative stress. For example, glycolytic enzymes (*e.g.*, GAPDH, pyruvate kinase, aldolase, phosphoglycerate kinase, triose phosphate isomerase), TCA cycle enzymes (*i.e.*, aconitase, KGDH, IDH3, succinyl-CoA transferase), and mitochondrial OXPHOS protein complexes (Complex I–V) can be glutathionylated, which results in activation or inactivation of their enzymatic activities thus modulating cellular energetics (12, 18, 25, 29, 44, 59, 70).

By contrast, enzyme-driven S-glutathionylation is typically specific and reversible, and is highly controlled by fluctuation in local GSH/GSSG pools (58, 79). The glutathione-S-transferase is the major enzyme mediating S-glutathionylation by adding GSH to cysteinyl residues of a protein producing PSSG (98); and Grx1 and Grx2 are the primary enzymes catalyzing the reversible reaction (58). To date, little is known about whether glutathionylation of proteins, particularly metabolic enzymes, can occur under reductive stress (high GSH/GSSG) and how this modification impairs cellular metabolic activities. These research topics deserve future investigation.

## Abbreviations Used

α-KG: α-ketoglutarate
6PGD: 6-phosphogluconate dehydrogenase
AGC1: *aspartate-glutamate carrier 1*
Ang II: angiotensin II
AOA: aminooxyacetic acid
DHAP: dihydroxyacetone phosphate
eIF2α: eukaryotic initiation factor 2α
ER: endoplasmic reticulum
F6P: fructose-6-phosphate
FH: fumarate hydratase
FTHFDH: 10-formyl-tetrahydrofolate dehydrogenase
G-3-P: glyceraldehyde-3-phosphate
G-6-P: glucose-6-phosphate
G6PD: glucose-6-phosphate dehydrogenase
GAPDH: glyceraldehyde-3-phosphate dehydrogenase
GCL: γ-glutamylcysteine ligase
GGT: γ-glutamyl transpeptidase
GLS: glutaminase
GLUD: glutamate dehydrogenase
GOT: glutamate-oxaloacetate transaminase
GPDH: glycerol-3-phosphate dehydrogenase
GPx: glutathione peroxidase
GR: glutathione reductase
Grx: glutaredoxin
GS: GSH synthetase
GSH: glutathione
GSSG: GSH disulfide
H_2_O_2_: hydrogen peroxide
HIF-1α: hypoxia-inducible factor 1α
Hsp27: heat shock protein 27
IDH: isocitrate dehydrogenase
IR: ischemia/reperfusion
KGDH: α-ketoglutarate dehydrogenase
LDH: lactate dehydrogenase
L2HG: l-2-hydroxyglutarate
MDH2: malate dehydrogenase 2
ME: malic enzyme
MTHFD: methylene-tetrahydrofolate dehydrogenase
NAC: *N*-acetyl-l-cysteine
NAD^+^: nicotinamide adenine dinucleotide
NADH: reduced NAD^+^
NADK: NAD^+^ kinase
NADP^+^: phosphorylated NAD^+^
NADPH: reduced NADP^+^
NAM: nicotinamide
NNT: nicotinamide nucleotide transhydrogenase
NR: nicotinamide riboside
NOX: NAD(P)H oxidase
NRF2: nuclear factor (erythroid-derived 2)-like 2
O_2_^•−^: superoxide anion
OAA: oxaloacetate
OXPHOS: oxidative phosphorylation
PDH: pyruvate dehydrogenase
PHD2: prolyl hydroxylase domain-containing protein 2
PPP: pentose phosphate pathway
Prx: peroxiredoxin
PSSG: protein-glutathione disulfide
ReBC: redox buffer capacity
RNS: reactive nitrogen species
ROS: reactive oxygen species
SDH: succinate dehydrogenase
SIRT: *sirtuin* deacetylase
SOD: superoxide dismutase
TCA: tricarboxylic acid
THF: tetrahydrofolate
TR: thioredoxin reductase
Trx: thioredoxin
xCT: cystine transporter
